# Effect of grinding, extraction time and type of coffee on the physicochemical and flavour characteristics of cold brew coffee

**DOI:** 10.1038/s41598-019-44886-w

**Published:** 2019-06-11

**Authors:** Nancy Cordoba, Laura Pataquiva, Coralia Osorio, Fabian Leonardo Moreno Moreno, Ruth Yolanda Ruiz

**Affiliations:** 1 Universidad de La Sabana, Faculty of Engineering, Doctorado en Biociencias, Campus Universitario del Puente del Común, Km 7 Autopista Norte de Bogota, Chía-Cundinamarca, Colombia; 2 Universidad de La Sabana, Faculty of Engineering, Maestría en Diseño y Gestión de Procesos, Campus Universitario del Puente del Común, Km 7 Autopista Norte de Bogota, Chía-Cundinamarca, Colombia; 3 Universidad de La Sabana, Faculty of Engineering, Grupo de Investigación en Procesos Agroindustriales, Campus Universitario del Puente del Común, Km 7 Autopista Norte de Bogota, Chía-Cundinamarca, Colombia; 4 Departamento de Química, Universidad Nacional de Colombia-Sede Bogotá, AA 14490 Bogotá, Colombia

**Keywords:** Chemical engineering, Chemical engineering

## Abstract

The effects of grinding (medium-coarse) and extraction time (14–22 h) on the physicochemical and sensorial properties of cold brew coffee produced using two types of Colombian specialty coffees (Huila and Nariño) were evaluated. Cold coffee brewed under coarse grinding and 22 h of extraction exhibited the highest values of total dissolved solids, extraction yield, pH, titratable acidity (TA), and total phenolic content. The type of coffee used mainly affected the TA and pH. All cold brew coffee samples had lower TA values than their hot counterparts. Nariño cold brew samples had higher TA values than those of Huila in all treatments evaluated. Higher scores were reported in the sensorial evaluation of cold brew coffee when prepared using the shortest time (14 h) and coarse grinding for both coffee types. These coffees were characterized by strong sweetness, fruity and floral flavours, medium bitterness and acidity, and a creamy body. Furans, pyrazines, ketones, aldehydes, pyrroles, esters, lactones, furanones, and phenols were detected as odour-active compounds. The findings of this study demonstrate that the particle size, contact time, and coffee type affect the physicochemical and sensorial characteristics of cold brew coffee, leading to cold brew coffees with different flavour profiles.

## Introduction

Every day, approximately 2.25 billion cups of coffee are consumed globally^1^. The popularity of coffee arises from the stimulating effects of caffeine and from its pleasant aroma and flavour and, more recently, from its health benefits^2–4^. The characteristic coffee aroma and flavour are related to the volatile and non-volatile compounds generated during roasting that are brought to the cup by means of brewing^5^. Several metabolites present in the green beans during the harvesting and post-harvesting process contribute to the flavour after roasting and are associated with the quality of the coffee brew flavour. Since the 1990s, coffee quality has been related to the term “specialty coffee” or “café gourmet”, which refer to the highest quality of green coffee beans, with an excellent and unique flavor^6^. These beans have a known geographical origin and mostly include certified beans^7^.

The majority of coffee beverages consumed are prepared by various hot brewing methods, depending on the geographic, cultural, and social context, as well as on personal preferences^8–10^. From an engineering viewpoint, coffee brewing is considered a solid-liquid extraction, where the roasted and ground coffee is in intimate contact with water. The water acts as a solvent to extract the water-soluble compounds, and depending on the extraction technique used, insoluble compounds may also be present in the extraction water as dissolved or suspended solids^11^. Finally, volatile and non-volatile flavour compounds are extracted from the ground coffee and are dispersed in the final coffee beverage^12^. In this process, the size reduction of the roasted beans by grinding is a prerequisite for the controlled extraction and dispersion of the chemical compounds in the coffee brew. Grinding is an operation that breaks down the roasted coffee into particles or smaller fragments. The formation of small particles, with a large particle surface area, is essential for the rapid liberation of carbon dioxide, the reduction of the diffusion distance for soluble substances during extraction, and for the improved transfer of colloidal substances to the liquid phase^13^.

Due to the relevance of chemicals to the sensory characteristics of the coffee beverage, recent studies have focused on evaluating the effects of several variables along the entire production chain. In the literature, the most studied hot extraction methods are the espresso and filtered coffee methods. Studies have been carried to evaluate the influence of the product formulation (e.g., bean botanical type, post-harvest processes, roasting degree) and brewing process variables (extraction time, flow rate, temperature, particle size distribution, and water pressure) on the physicochemical attributes and sensory profiles^5,8,9,14–16^.

Numerous methods have emerged for coffee brewing, and most of them use high-temperature water (near the boiling point) and short times that do not exceed 5 minutes. However, in recent years, a new method has gained popularity in the market—the method of cold extraction. Cold brewing is carried out at room temperature (20 to 25 °C or colder) over a longer period than traditional hot brewing methods, with typical steeping times ranging from 8 to 24 hours^17^. Cold brew coffee can be made by dripping, direct or indirect immersion or by the French press method^10^.

Low temperatures and long contact times produce a final coffee beverage with different physicochemical and sensory characteristics. These parameters affect the extraction speed and way of the chemical compounds present in the roasted coffee. The chemical compounds present in the coffee exhibit different chemical properties (e.g., polarity and solubility); therefore, they have different extraction kinetics^15,18^. Overall, a higher temperature increases the solubility^19^, and it affects the saturated vapour pressure of aroma compounds. Higher temperatures lead to a greater evaporation of organic volatile compounds (VOCs) and consequently to a higher release of these compounds. In cold extraction at room temperature, Fuller and Rao^17^ observed that the concentrations of 3-chlorogenic acid (3-CGA) and caffeine increased rapidly over the first 180 minutes before slowing and reaching equilibrium at approximately 400 minutes for different degrees of coffee roasting and grinding^17^.

Hot extraction has been widely studied, and it is well known that this brewing method affects the polyphenol extraction, caffeine content, total solids content, antioxidant activity, and the volatile compound profile^20^. Several studies^8,9,16,21^ have compared different hot extraction methods and the physicochemical attributes and sensory profiles of these coffee brews. Conversely, despite the growing consumption of cold brew coffee and the interest in this coffee market, to the best of the authors’ knowledge few scientific studies regarding its physicochemical and sensory attributes have been published.

Regarding the cold extraction conditions and the final cold beverage composition, Kim and Kim^22^ found significant differences in the concentrations of non-volatile components such as caffeine, chlorogenic acid and trigonelline and in the flavour profile when they evaluated different extraction methods (dripping vs. stepping) and times (3,6,9 and 18 h). Higher concentrations of non-volatiles were found in the stepping method^22^. Non-volatile compounds have also been studied by Fuller and Rao^17^, who analysed the extraction kinetics and equilibrium concentrations of caffeine and 3-chlorogenic acid (3-CGA) in cold brew coffee compared with hot methods (French press) under different roast degree and grinding conditions. Higher concentrations of 3-CGA and caffeine were observed in cold brew coffee made with medium-roasted beans, and these compounds reached equilibrium at between 6 and 7 h of extraction, while 3-CGA concentrations and the pH were comparable between the cold and hot brews^17^. On the other hand, Lane and coworkers^23^ found that the caffeine concentration in cold brew coffee did not exhibit major differences compared to that of hot coffee^23^. Recently, Angeloni *et al*.^24^ measured the caffeine and cinnamic acid concentrations and some physicochemical and sensory characteristics in cold brew coffees that were prepared by using two cold brew methods. They reported that a higher temperature (22 °C) increased the total solids content, and the caffeine, caffeoylquinic acid, and 5-chlorogenic acid concentrations compared to the extractions carried out at 5 °C. Similarly, they found differences between the cold brew methods and temperatures evaluated regarding the flavour profiles, as measured in terms of bitterness, sweetness, sourness, and global intensity^24^.

Due to more recent investigations that have indicated the potential health benefits of coffee beverage intake^25^, some studies on cold brew coffee have evaluated bioactive compounds. For instance, Shin^26^ studied the chemical characteristics of polysaccharides isolated from cold brew coffee and found that cold brew coffee extracts could improve the functions of macrophages and the intestinal immune system. In the last year, Rao and Fuller^27^ evaluated the acidity and antioxidant activity of cold brew coffee made from light roast coffees of different coffee producing countries. They found that cold brew coffee extracts had lower concentrations of acidic compounds than hot brew coffee extracts prepared from the same beans and that the hot brew coffees had higher antioxidant capacities than their cold brew counterparts^27^.

Cold brew coffee is a growing market within the coffee industry. In recent years, scientific interest has increased in this field. However, the effects of the cold extraction conditions on the chemical composition (non-volatile and volatile compounds) and sensory characteristics of cold brew coffee are still being studied. Therefore, this work analysed the extraction variables of extraction time, type of specialty coffee (Nariño and Huila), and grinding using cold brewing by the indirect immersion method. A sensory analysis was carried out to select the cold drinks most appreciated for their flavour compared with their hot counterparts. The selected cold brew coffees were analysed to determine their volatile organic compounds. This experimental configuration combining instrumental analysis with sensory evaluation to determine the volatile compounds and flavour characteristics of cold brew coffee has not yet been reported.

## Results

### Physicochemical characteristics of cold brew coffee under different extraction conditions

**Figure 1:**
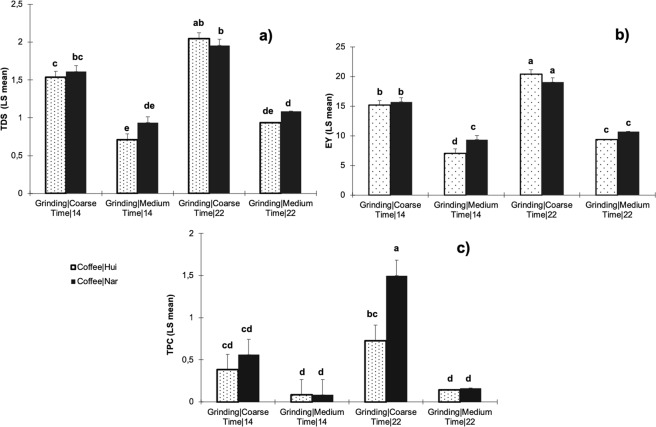
Physicochemical characteristics of the cold brew coffees (**a**) Total Dissolved Solids-TDS (%), (**b**) Extraction yield EY (%) and (**c**) Total Phenolic Content-TPC (g/L gallic acid) under different grinding (coarse and medium), contact time (14 and 22 h) and type of specialty coffees (Hui: Huila and Nar: Nariño). Different letters indicate statistically significant differences (p < 0.05) among treatments.

**Figure 2:**
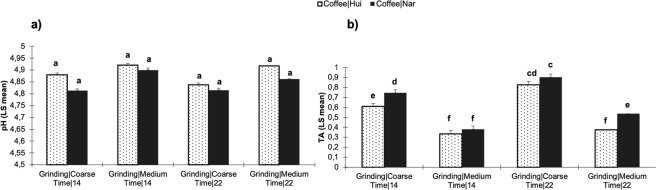
Physicochemical characteristics of the cold brew coffees: (**a**) pH and (**b**) total titratable acidity-TA (mg/g chlorogenic acid) under different grinding (coarse and medium), contact time (14 and 22 h) and type of specialty coffees (Hui: Huila and Nar: Nariño). Different letters indicate statistically significant differences (p < 0.05) among treatments.

The physicochemical characteristic results of cold brewed coffee under different extraction condition (grinding, time and type of coffee) are shown in Figs 1 and 2. The process variables of grinding and contact time were statistically significant (p < 0.05) for extraction yield (EY), total dissolved solids (TDS), total phenolic content (TPC), pH and total titratable acidity (TA), while the type of specialty coffee used was only statistically significant (p < 0.05) for pH and TA. According to the coefficients of the statistical analysis, grinding was the factor with the greatest impact on the physicochemical parameters of the cold brew coffee, followed by the extraction time. A coarse grinding and a long extraction time (22 h) increased of the EY percentage and TDS for both coffees evaluated (Fig. 1a,b). Grinding and time were statistically significant (p < 0.05) for the phenolic content (TPC) of the cold brew coffee. A higher TPC was found when using coarse grinding and 22 h of extraction with Nariño coffee (1.50 ± 0.37 g/L gallic acid). Although the type of coffee was not statistically significant for the TPC (p **>** 0.05), Nariño cold brew samples showed higher values than Huila samples, mainly under coarse grinding (Fig. 1c). Polyphenols are present in coffee and have been related to human health. They have been recognized for their strong antioxidant properties^28^. Publications regarding cold brew coffee and their phenolic content are scarce. Although chlorogenic acids and their related molecules have been associated primarily with the functional properties of coffee, recent studies in cold brewing using different origins of coffee have reported low total caffeoylquinic acid (CQA) concentrations and total antioxidant activities compared with their hot brew counterparts^27^.

Although the grinding level and time had a significant effect (p < 0.05) on the pH and TA, these parameters were also influenced by the type of coffee used for cold brewing. The lowest pH values (more acidic) for Nariño and Huila coffees were 4.81 ± 0.01 and 4.88 ± 0.0, respectively, which were obtained under coarse grinding and 14 h of extraction for both coffees. Overall, higher pH values (less acidic) were reported for Huila coffee (4.92 ± 0.02), while the higher pH value from Nariño cold brew was 4.90 ± 0.0. Recently, Fuller and Rao^17^ reported pH values between 5.40 and 5.63 for cold brew *Coffea arabica* (Kona Typica)^17^. In addition, Rao and Fuller^27^ found that the pH values of cold brew coffees of different coffee types (origin countries) ranged between 4.96 and 5.13^27^. Regarding total titratable acidity, the highest values (0.90 ± 0.04 mg/g chlorogenic acid) were found in the coarse ground and 22 h cold brew samples using Nariño coffee. Lower TA values (0.33 ± 0.03 mg/g chlorogenic acid) were identified in cold brew coffee with medium grinding and 14 h of extraction; these values were lower when Huila coffee was used.

### Comparison of physicochemical characteristics between cold and hot coffee brews

Commercial vendors and coffee enthusiasts often suggest that cold brew and hot brew coffees have different taste profiles, acidity levels, and caffeine contents^17,27^. In this work, physicochemical characteristics of cold and hot brews were evaluated when prepared using a similar ratio of coffee/water. The results are shown in Table 1. Cold brew coffees of both specialty coffee types exhibited higher EY and TDS values than their hot counterparts, mainly when coarse grinding and long extraction times (22 h) were used.

**Table 1:** Comparison of physicochemical characteristics using cold and hot brew methods TDS: Total dissolved solids, EY: Extraction yield, TPC: Total Phenolic Content, TA: total titratable acidity. (mean ± 95%, confidence interval, n = 6).

Brewing method	Extracton conditions	TDS (%)	EY (%)	pH	TPC (g/L gallic acid)	TA (mg/g chlorogenic acid)
Hot brewing	Hot-Nariño	1.87 ± 0.00^a^	15.20 ± 0.00^b^	4.44 ± 0.11^b^	1.36 ± 0.14^a^	2.00 ± 0.06^a^
	Hot-Huila	1.83 ± 0.00^a^	14.90 ± 0.01^b^	4.36 ± 0.09^b^	1.24 ± 0.00^a,b^	1.81 ± 0.03^b^
Cold brewing	Coarse-14h-Huila	1.54 ± 0.00^c^	15.21 ± 0.01^b^	4.88 ± 0.01^a^	0.38 ± 0.09^c,d^	0.61 ± 0.01^e^
	Coarse-14h-Nariño	1.61 ± 0.00^b,c^	15.73 ± 0.01^b^	4.81 ± 0.01^a^	0.56 ± 0.04^c,d^	0.75 ± 0.05^d^
	Medium-14h-Huila	0.71 ± 0.00^e^	7.06 ± 0.00^d^	4.92 ± 0.01^a^	0.08 ± 0.03^d^	0.33 ± 0.03^f^
	Medium-14h-Nariño	0.94 ± 0.00^d,e^	9.36 ± 0.01^c^	4.90 ± 0.01^a^	0.08 ± 0.03^d^	0.38 ± 0.02^f^
	Coarse-22h-Huila	2.04 ± 0.00^a,b^	20.39 ± 0.00^a^	4.84 ± 0.00^a^	0.73 ± 0.35^b,c^	0.83 ± 0.01^c,d^
	Coarse-22h-Nariño	1.96 ± 0.00^b^	19.05 ± 0.01^a^	4.82 ± 0.01^a^	1.50 ± 0.37^a^	0.90 ± 0.04^c^
	Medium-22h-Huila	0.94 ± 0.00^d,e^	9.40 ± 0.01^c^	4.92 ± 0.02^a^	0.14 ± 0.01^d^	0.37 ± 0.03^f^
	Medium-22h-Nariño	1.09 ± 0.00^d^	10.72 ± 0.00^c^	4.86 ± 0.01^a^	0.16 ± 0.01^d^	0.54 ± 0.03^e^

Different letters (a, b, c, d, e, f) in the same column indicate statistically significant differences (*p* < 0.05) among treatments.

Zanoni *et al*.^29^ found a higher content of total solids and caffeine with a prolonged extraction time under hot extraction. A longer brewing time allows increased extraction of some compounds^9^, but these compounds can be highly susceptible to oxidation and degradation^30^.

Hot coffee brews showed significant differences between them regarding TA and TPC. Higher values for these variables were found in the brews made with Nariño coffee (2.00 ± 0.06 mg/g chlorogenic acid for TA and 1.36 g/L gallic acid for TPC). In cold brew coffee, the highest TPC values were found in treatments that used Nariño coffee, coarse grinding and 22 h of extraction (1.50 ± 0.37 g/L gallic acid). This value was comparable with the TPC found in the hot brews (1.36 ± 0.14 g/L gallic acid) made with the same coffee. The differences in the TPC values can be explained because there are several water-soluble polyphenols present in roasted coffee which exhibit different chemical properties, some of them such as chlorogenic acids, caffeine, and soluble melanoidins are removed under different contact times^31^. Besides, the concentration of these compounds in roasted coffee beans vary owing to the coffee’s origin, post-harvest processing, roasting, and storage^32^. These facts could explain the difference in the phenolic concentration observed between types of coffees and extractions ways.

In this study, the pH values of the cold brew coffees were significantly different (p < 0.05) from the hot brews. Cold brew coffees exhibited higher pH values (less acidic) than their hot counterparts. In the hot beverages, the total acidity was higher than in all of the cold brew coffees. Total acidity in the hot beverages was higher than in the cold brew coffee. Recently, Rao and Fuller^27^ reported that the titratable acidity in hot coffee extracts was larger than that measured in cold brew coffee extracts, which could indicate that hot brewing was able to extract more acids and additional acidic compounds^27^. Moreover, several studies have revealed that the pH and TA are dependent on many factors, such as the geographic origin of the green coffee beans, fruit maturity, harvesting processes, weather conditions during harvesting and drying, and all steps in the post-harvest processing. Furthermore, pH and acidity have been associated with the type of roasting process and brewing method^28,33,34^.

### Flavour profile of cold and hot brew coffees

In this study, the flavour profile of the coffee brew was evaluated regarding its general characteristics and the intensity of specific attributes that are commonly used in the coffee industry. Global characteristics include terms such as fragrance and aroma, acidity, body, flavour, and aftertaste, while the attributes are specific descriptors that may not occur in every cup of coffee, but when they are present can add complexity and uniqueness to the coffee^35^. The results from the sensory analysis for the cold and hot brew coffees are shown in Tables 2 and 3.

**Table 2:** Comparison of global sensory characteristics using cold and hot brew methods (mean ± 95% Confidence Interval, n = 6).

Brewing method	Extracton conditions	Aroma	Taste	Aftertaste	Acidity	Body	Overall impact
Hot brewing	Hot brew-Nar	3.7 ± 0.76^a^	4.0 ± 0.50^a^	4.0 ± 0.50^a^	3.0 ± 0.0^a^	4.0 ± 0.50^a^	4.5 ± 0.00^a^
	Hot brew-Hui	3.5 ± 0.00^a,b^	4.0 ± 0.00^a^	3.8 ± 0.58^a^	3.0 ± 0.00^a^	4.0 ± 0.00^a^	4.3 ± 0.29^a,b^
Cold brewing	Coarse-14h-Hui	2.3 ± 0.58^c,d^	2.5 ± 0.00^b,c^	2.5 ± 0.50^b^	1.7 ± 0.76^b^	2.2 ± 0.29^b,c^	3.8 ± 0.29^b,c^
	Coarse-14h-Nar	2.7 ± 0.29^c,d^	3.0 ± 0.00^b^	2.5 ± 0.50^b^	1.5 ± 0.00^b,c^	2.3 ± 0.76^b^	4.3 ± 0.29^a,b^
	Medium-14h-Hui	2.0 ± 0.50^c,d^	2.0 ± 0.50^c^	1.5 ± 0.08^c^	1.0 ± 0.00^c^	1.0 ± 0.00^d^	2.5 ± 0.50^e^
	Medium-14h-Nar	2.8 ± 0.29^b,c^	2.8 ± 0.58^b^	2.0 ± 0.50^b,c^	1.5 ± 0.00^b,c^	1.7 ± 0.29^c^	3.3 ± 0.29^c,d^
	Coarse-22h-Hui	2.5 ± 0.00^c,d^	2.5 ± 0.00^b,c^	2.5 ± 0.00^b^	1.5 ± 0.50^b,c^	2.00 ± 0.00^b,c^	3.7 ± 0.29^c,d^
	Coarse-22h-Nar	2.7 ± 0.29^c,d^	2.8 ± 0.58^b^	2.2 ± 0.58^b,c^	1.2 ± 0.29^b,c^	2.00 ± 0.00^b,c^	3.8 ± 0.29^b,c^
	Medium-22h-Hui	2.5 ± 0.50^c,d^	2.0 ± 0.00^c^	1.7 ± 0.29^c^	1.2 ± 0.50^b,c^	1.8 ± 0.58^b,c^	3.2 ± 0.29^d^
	Medium-22h-Nar	2.7 ± 0.29^c,d^	2.7 ± 0.29^b^	1.8 ± 0.58^b,c^	1.2 ± 0.29^b,c^	2.2 ± 0.29^b,c^	3.8 ± 0.29^b,c^

Different letters (a, b, c, d) indicate statistically significant differences (p < 0.05) in the global sensory characteristics for each treatment (columns).

**Table 3:** Comparison of sensory attributes using cold and hot brew methods (mean ± 95% confidence Interval, n = 6).

Global Characteristics	Atributte	Hot brew Nariño	Hot brew Huila	Coars14h-Huila	Coarse-14h-Nariño	Medium-14h-Huila	Medium-14h-Nariño	Coarse-22h-Huila	Coarse-22h-Nariño	Medium-22h-Huila	Medium-22h-Nariño
Aroma	Malt	0.50 ± 0.6^d^	0.67 ± 0.8^c,d^	1.00 ± 1.3^b,c,d^	**2.67 ± 0.8** ^**a**^	1.50 ± 1.4^a,b,c,d^	1.83 ± 0.8^a,b,c^	1.66 ± 1.0^a,b,c,d^	2.00 ± 0.9^a,b^	2.00 ± 0.9^a,b^	1.83 ± 1.2^a,b,c^
	Pepper	1.00 ± 0.9^a,b^	0.33 ± 0.5^b^	0.83 ± 1.0^a,b^	0.83 ± 1.0^a,b^	0.17 ± 0.4^b^	**1.33 ± 0.8** ^**a**^	0.67 ± 0.8^a,b^	0.33 ± 0.5^b^	0.17 ± 0.4^b^	0.66 ± 1.0^a,b^
	Almonds	1.33 ± 1.2^a,b^	1.00 ± 0.9^a,b^	1.00 ± 1.1^a,b^	0.83 ± 1.0^b^	1.67 ± 1.4^a,b^	**2.33 ± 1.2** ^**a**^	2.17 ± 1.3^a,b^	1.17 ± 1.3^a,b^	1.67 ± 1.0^a,b^	1.50 ± 1.5^a,b^
	Dark chocolate	**2.33 ± 0.5** ^**a**^	2.00 ± 1.4^a,b^	1.67 ± 1.4^a,b^	1.17 ± 1.2^a,b^	0.67 ± 0.8^b^	1.33 ± 1.2^a,b^	1.17 ± 1.2^a,b^	1.17 ± 1.0^a,b^	1.83 ± 1.5^a,b^	1.33 ± 1.2^a,b^
	White chocolate	**1.50 ± 1.2** ^**a**^	0.83 ± 1.0^a,b^	0.50 ± 0.8^a,b^	0.33 ± 0.5^b^	0.67 ± 0.8^a,b^	1.00 ± 1.0^a,b^	0.67 ± 1.0^a,b^	0.50 ± 0.8^a,b^	0.33 ± 0.8^b^	1.00 ± 1.3^a,b^
	Vanilla	2.33 ± 1.4^a,b^	**2.50 ± 0.5** ^**a**^	1.17 ± 1.2^b,c^	1.00 ± 0.9^c^	0.83 ± 0.8^c^	1.17 ± 1.1^b,c^	1.67 ± 1.0^a,b,c^	0.83 ± 1.0^c^	0.83 ± 1.2^c^	0.83 ± 1.2^c^
	Honey	2.67 ± 1.9^a,b^	**3.00 ± 2.0** ^**a**^	1.33 ± 1.4^a,b^	2.33 ± 1.5^a,b^	1.00 ± 1.3^b^	1.83 ± 1.5^a,b^	1.50 ± 1.6^a,b^	1.33 ± 1.2^a,b^	2.00 ± 1.3^a,b^	1.50 ± 1.4^a,b^
	Cocoa	0.83 ± 0.4^c^	2.00 ± 1.3^a,b,c^	1.67 ± 1.0^a,b,c^	1.33 ± 1.0^b,c^	1.33 ± 0.8^b,c^	**2.83 ± 1.1** ^**a**^	1.67 ± 0.8^a,b,c^	2.17 ± 1.3^a,b^	2.170 ± 1.2^a,b^	2.67 ± 0.5^a^
	Black fruity	1.50 ± 1.2^a,b,c^	1.83 ± 1.4^a,b^	0.67 ± 0.8^b,c,d^	**2.00 ± 1.3** ^**a**^	0.17 ± 0.4^d^	0.83 ± 1.2^a,b,c,d^	0.50 ± 0.5 ^c,d^	1.83 ± 1.5^a,b^	1.17 ± 1.0^a,b,c,d^	0.83 ± 1.0^a,b,c,d^
Taste	Malt	0.67 ± 1.2^b^	0.50 ± 0.5^b^	1.67 ± 1.6^a,b^	1.67 ± 1.0^a,b^	1.67 ± 1.4^a,b^	1.33 ± 1.1^a,b^	1.33 ± 1.5^a,b^	1.17 ± 1.0^a,b^	1.00 ± 1.1^a,b^	**2.17 ± 0.4** ^**a**^
	Almonds	1.50 ± 1.5^a,b^	2.17 ± 1.2^a^	1.50 ± 1.2^a,b^	1.83 ± 1.6^a,b^	0.67 ± 0.8^b^	**2.33 ± 1.2** ^**a**^	1.83 ± 1.2^a,b^	1.67 ± 0.8^a,b^	1.17 ± 1.2^a,b^	1.33 ± 1.2^a,b^
	Dark chocolate	**3.00 ± 1.5** ^**a**^	2.33 ± 1.5^a,b^	2.50 ± 1.8^a,b^	2.00 ± 1.7^a,b,c^	1.00 ± 0.9^b,c^	1.33 ± 1.6^a,b,c^	1.83 ± 1.8^a,b,c^	2.00 ± 1.7^a,b,c^	0.50 ± 0.5^c^	1.33 ± 1.5^a,b,c^
	White chocolate	1.67 ± 1.9^a,b^	2.17 ± 1.6^a^	1.00 ± 1.1^a,b^	1.50 ± 1.9^a,b^	0.50 ± 0.5^b^	0.50 ± 1.3^b^	0.33 ± 0.5^b^	0.33 ± 0.5^b^	0.83 ± 1.0^a,b^	0.83 ± 1.2^a,b^
	Floral	0.67 ± 1.2^b,c^	0.50 ± 1.2^c^	2.33 ± 1.6^a,b^	2.00 ± 1.8^a,b,c^	1.50 ± 1.6^a,b,c^	**2.67 ± 1.6** ^**a**^	1.50 ± 1.5^a,b,c^	**2.67 ± 1.2** ^**a**^	1.00 ± 0.9^a,b,c^	2.17 ± 1.3^a,b,c^
	Cocoa	2.50 ± 0.8^a,b^	**3.33 ± 0.8** ^**a**^	1.67 ± 1.0^b,c^	1.83 ± 1.6^b,c^	1.17 ± 1.0^b,c^	2.33 ± 1.3^a,b,c^	2.50 ± 1.4^a,b^	1.67 ± 1.4^b,c^	1.00 ± 1.1^c^	2.00 ± 1.7^a,b,c^
	Red fruity	1.83 ± 1.6^a,b,c^	2.83 ± 0.8^a,b^	1.17 ± 1.3^b,c^	**3.33 ± 1.6** ^**a**^	0.83 ± 0.8^c^	1.67 ± 1.4^a,b,c^	1.50 ± 1.6^b,c^	2.33 ± 1.9^a,b,c^	2.00 ± 1.3^a,b,c^	1.50 ± 1.5^b,c^
	Citric fruity	0.83 ± 0.8^a,b,c^	**2.50 ± 2.1** ^**a**^	0.50 ± 0.8^b,c^	2.00 ± 1.5^a,b^	1.33 ± 1.8^a,b,c^	1.33 ± 1.7^a,b,c^	1.33 ± 1.8^a,b,c^	1.17 ± 2.0^a,b,c^	0.00 ± 0.0^c^	1.33 ± 2.2^a,b,c^
	Bitter	2.00 ± 0.9^a,b^	2.00 ± 0.9^a,b^	1.50 ± 1.0^a,b^	**2.17 ± 0.8** ^**a**^	1.00 ± 0.6^b^	2.00 ± 0.9^a,b^	1.67 ± 0.8^a,b^	**2.17 ± 1.5** ^**a**^	1.17 ± 0.8^a,b^	1.50 ± 0.5^a,b^
	Astringent	**1.83 ± 1.8** ^**a**^	0.67 ± 0.8^a,b^	0.50 ± 0.8^b^	1.33 ± 1.5^a,b^	0.33 ± 0.5^b^	1.00 ± 1.1^a,b^	1.00 ± 0.9^a,b^	0.67 ± 1.2^a,b^	0.33 ± 0.5^b^	0.83 ± 1.2^a,b^
Aftertaste	Bitter	2.50 ± 0.5^a^	2.00 ± 0.6^a,b^	2.33 ± 1.0^a,b^	**2.67 ± 0.8** ^**a**^	2.00 ± 0.9^a,b^	2.17 ± 1.0^a,b^	2.00 ± 0.9^a,b^	**2.67 ± 1.2** ^**a**^	1.33 ± 1.0^b^	1.67 ± 0.5^a,b^
	Astringent	1.50 ± 1.2^a,b^	1.17 ± 1.3^a,b^	1.50 ± 1.9^a,b^	**2.00 ± 1.7** ^**a**^	0.33 ± 0.5^b^	1.17 ± 1.1^a,b^	0.83 ± 1.3^a,b^	0.67 ± 1.0^a,b^	0.33 ± 0.5^b^	1.00 ± 1.3^a,b^
	Sweet	3.17 ± 1.2^a,b^	3.17 ± 0.8^a,b^	**4.33 ± 0.8** ^**a**^	3.50 ± 1.0^a,b^	3.00 ± 1.1^b^	3.00 ± 1.1^b^	3.83 ± 1.3^a,b^	3.17 ± 1.2^a,b^	4.00 ± 1.3^a,b^	3.00 ± 0.9^b^
	Long-after taste	3.17 ± 1.0^a,b,c^	3.83 ± 1.0^a,b^	2.83 ± 1.6^a,b,c,d^	3.50 ± 1.0^a,b,c^	1.50 ± 0.5^d^	2.50 ± 1.3^b,c,d^	**4.00 ± 1.1** ^**a**^	3.50 ± 1.0^a,b,c^	2.33 ± 1.8^c,d^	2.67 ± 1.6^a,b,c,d^
Acidity	Medium	3.0 ± 0.00^a^	**3.0 ± 0.00** ^**a**^	1.7 ± 0.76^b^	1.5 ± 0.00^b,c^	1.0 ± 0.00^c^	1.5 ± 0.00^b,c^	1.5 ± 0.50^b,c^	1.2 ± 0.29^b,c^	1.2 ± 0.50^b,c^	1.2 ± 0.29^b,c^
Body	Smooth body	2.33 ± 1.9^a,b,c^	2.00 ± 1.1^a,b,c^	**3.17 ± 0.8** ^**a**^	2.83 ± 1.0^a,b,c^	1.67 ± 0.8^b,c^	3.00 ± 1.3^a,b^	1.67 ± 1.4^b,c^	1.67 ± 1.0^b,c^	1.50 ± 1.4^c^	2.83 ± 1.7^a,b,c^
	Creamy body	2.17 ± 1.8^a,b,c^	2.50 ± 1.0^a,b^	2.33 ± 1.6^a,b^	**3.33 ± 0.8** ^**a**^	0.83 ± 0.8^c^	1.33 ± 1.4^b,c^	2.33 ± 1.5^a,b^	2.17 ± 1.2^a,b,c^	2.17 ± 1.2^a,b,c^	3.00 ± 1.4^a^

Different letters (a,b,c,d) indicate statistically significant differences (p < 0.05) in the flavor attribute in each treatments (rows). Scores in bold represent the treatment with the highest value for each flavor attribute.

Regarding global flavour characteristics, the data in Table 2 indicate significant differences (p < 0.05) and higher scores in the taste, aroma, aftertaste, acidity, and body of hot brews compared with all cold brew coffee treatments. However, the overall impact of the cold brewed coffees had scores that were close to their hot brewed counterparts. The overall impact reflects the holistically integrated rating of the sample as perceived by the individual panellist^36^. Consequently, the assessors showed that although some flavour characteristics in cold brew coffees were less intense compared to the hot beverage, the taste of the cold drinks, according to their impression (overall impact), was acceptable for a coffee beverage. The best scores for cold brewed coffees in terms of overall impact were for those samples brewed using coarse grinding and 14 h of extraction for both coffee types.

The results in Table 3 show that cold brew coffee had higher scores in the malt, pepper, almond, cocoa, and black fruit aroma attributes than their hot counterparts. The malt aroma evidenced a higher score in all cold brew treatments and was significantly different (p < 0.05) than that in the hot brew treatments. On the other hand, the hot beverages presented a higher score in the aroma attributes related to chocolate, vanilla, and honey. The flavour profile of the cold brew coffee samples was characterized by a strong malt taste, and it was significantly different from their hot counterparts. Other flavour attributes such as almond, floral, red fruit, and bitter tastes were also evaluated and had high scores in the cold brew coffee. The aftertaste of the cold brew coffees exhibited higher scores in terms of bitterness, astringency, and sweetness than their hot counterparts. All cold brew samples made under medium grinding and 14 h of extraction gave lower scores for the long-aftertaste than those using hot brewing. The hot coffee samples exhibited a medium acidity, which was higher than and significantly different (p < 0.05) from the cold brew coffee. On the other hand, the cold brew samples were characterized by a creamy and smooth body; their scores in some cases were equal to or higher than the scores of the hot coffee brews.

Overall, cold brew coffee is typically considered to be sweet, with chocolate and syrupy notes^10^. In the scientific literature, there are limited data regarding the flavour profile of coffee brewed under different extraction conditions. Kim and Kim^22^ found differences in flavour in terms of chocolate, winey, acidity, sweetness, bitterness, body, and aftertaste attributes when stepping and dripping methods were used. They found that cold brew coffee at 18 h was the most acceptable to consumers according to the flavour profile^22^. Recently, Angeloni and coworkers^24^ reported that the flavour can vary depending on the cold brew method (dripping and immersion), but in general they found that cold brew coffees can exhibit higher intensities of sugar caramelization, sweetness, and bitterness^24^.

### Organic volatile compounds in cold brew coffee

**Figure 3:**
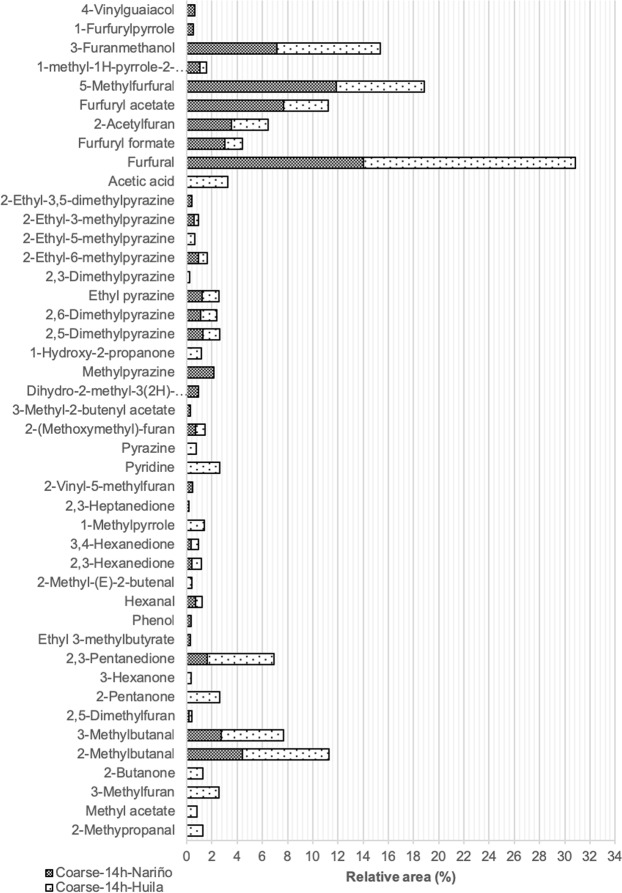
Relative percentage of volatile compounds from Nariño and Huila cold brew coffee samples with coarse grinding and 14 hours of extraction.

Flavour is a key attribute that defines the quality and the level of consumer acceptance of coffee products^37^. Thus, in the present study, the cold brew coffees samples with the best scores in the global sensorial characteristics were selected to determine their volatile compound profiles. The cold beverages prepared with Nariño and Huila coffees under coarse grinding conditions and 14 h of extraction were selected. The volatile compounds in the cold extracts were identified and quantified through GC–MS (Fig. 3). For the cold brew coffees, 30 volatile compounds were found in Nariño and 34 in Huila.

The volatile compound profiles were qualitatively and semi-quantitatively analysed (relation of area percentage from GC data). The highest numbers of volatile compounds (in order of abundance) were furans, pyrazines, ketones, aldehydes, pyrroles, esters, and acids for both cold brew coffee samples. Phenols were only found in the Nariño cold brew coffee, while two acids and pyridine were only detected in the Huila cold brew coffee. Regarding volatile compounds, Flament^18^ and Toci and Boldrin^38^ have reported that over 1000 volatile compounds have been identified thus far in roasted coffee. These can be divided into different classes, including (in order of abundance, with the approximate number of compounds) furans (150), pyrazines (100), phenols and ketones (90 in each class), pyrroles (80), hydrocarbons (76), carboxylic acids (60), esters (55), alcohols (50), and aldehydes (45). Clearly, not all of the approximately 1000 volatile compounds are relevant in terms of aroma. The literature suggests that only approximately 5% of these compounds may be responsible for the aroma of coffee, which would result in approximately 50 odorant compounds^38^ in roasted coffee. However, the presence of volatile compounds in the final coffee brew depends on the coffee bean chemical compounds, the roasting process, and the extraction technique. According to López-Galilea, *et al*.^39^, four classes of compounds appear to have a high impact in the aroma of coffee: pyrazines, furans, aldehydes, and ketones^39^. The current results showed that most of the volatile compounds detected in both of the cold coffee samples belonged to these chemical classes.

Figure 3 illustrates that furfural was the major volatile compound identified in the cold brew coffee for both specialty types. The highest value of furfural was from Huila coffee. Furfural is related to sweet, bread-like, and caramel flavours and is usually formed from glucose by thermal degradation^40^. In general, furans were the major class of volatile compounds detected in the cold brew coffee samples. Furans exhibit malty and sweet roasted aromas with relatively high sensory thresholds compared to other volatile groups found in coffee^32^. These characteristics could be related to the sensory attributes described by the assessors who found that the cold brew coffees had malt and sweet flavour attributes. Although similar compounds in the furans group were found in the two cold brew coffee beverages, the relative areas of certain compounds in the Nariño sample were higher, mainly for 5-methylfurfural (11.84% Nariño and 7.03% Huila), which has been associated with almond notes, caramel, and burnt sugar^41^.

Pyrazines were the group with the second highest number of volatile compounds in the two cold brew coffee beverages. Seven pyrazines were identified in the Nariño cold brew and eight in the Huila. Pyrazines are abundant in roasted coffee, have a low sensory threshold concentration and are of key importance to the flavour of coffee^12^. In general, pyrazines have been described as having nutty, earthy, roasty, and green aromas. They are most noticeable at higher serving temperatures^39^. Despite the low temperatures used during cold brewing, the samples analysed in this study showed flavour attributes associated with pyrazines when they were assessed by the judges. Ketones were also one of the three most abundant groups in both of the cold brew coffee samples. According to López-Galilea, *et al*.^39^, ketones are one of the most abundant compounds in filtered coffee brews. Seven ketones were identified in the Huila cold brew coffee, while only four were found in the Nariño cold brew. Ketones have been described as having buttery, caramel-like, musty, mushroom-like or fruity odour notes^39^. The cold brew coffee also presented aldehydes in their volatile profiles. 2-Methyl butanal and 3-methyl butanal showed high concentrations for both coffees (Huila and Nariño) (Fig. 3). These volatile compounds have been related to chocolate and malty odours in coffee^11,41^.

Pyrroles were also detected in the cold brew coffee. According to Falment^18^, these compounds have been reported in brewed coffee and are described as furan degradation products and amino acid derivatives^18^. Some pyrroles are known to be responsible for a peculiarly sweet and slightly flared smell in the coffee beverages^42^. There were also some esters present in the volatile fraction of the cold brew samples. These compounds have been described as strong odorants contributing to fruity notes^43^. The presence of esters in the cold brew coffee samples may be related to post-harvest processing. According to Fernandez-Alduenda^44^, methyl and ethyl esters are generated in semi-dry and natural processes, and they could be responsible for the fruity flavour^44^.

Acids and pyridines were only found in the cold brew from Huila coffee. Pyridines have been associated with low-quality coffee, as they are related to acidic, penetrating, and diffusive odour notes^45^. Acetic acid was identified in the Huila cold brew samples. This compound has been related to sharp, pungent, sour, and vinegar-like odours^46^. Acetic acid can be considered as an off-flavour when it is overpowering in coffee beverages^44^.

Phenols were found in the Nariño cold brew coffee. Based on both their high odour activity values and odour quality, phenols such as guaiacol and vinyl guaiacol may be responsible for the smoky phenolic odour notes in roasted coffee^47^. Additionally, they have been reported as being responsible for phenolic, spicy, and burnt aromas/flavours^21^. Among other compounds, dihydro-2-methyl-3 (2H)-furanone was only presented in the Nariño cold brew coffee (Fig. 3). Furanones are generated in coffee via the Maillard reaction and subsequent aldol condensation^32^ in the roasting process. Furanones are believed to be responsible for the sweet caramel aroma^32^, a characteristic attribute that was detected in the cold brew coffee sensory analysis.

Finally, many of the compounds identified in the cold brew drinks are characteristic of hot coffee beverages, with certain changes in concentration owing to the temperature and time conditions applied during the cold extraction process. The extraction rate of the chemical compounds is directly correlated with the polarity of the compounds, and the superior quality aroma of coffee beverage appears to be associated with well-defined temperature and pressure parameters, such as those established for espresso coffee beverages. Therefore, in hot coffee beverages, the compounds percolated in the greatest abundance will be those with higher polarity, such as carboxylic acids, alcohols, ketones, aldehydes, and pyrazines, all with smaller carbon chains^38^. These compounds diffuse rapidly in the nasal cavity, contributing to the potency and intensity of the coffee aroma in hot beverages.

## Discussion

In cold extraction, the grinding level had a significant effect on the physicochemical properties of the resulting coffee brews. The results showed that coarse grinding increased the TDS, EY and TPC values. However, it is well known that in a solid-liquid extraction, finer grinds enhance the extraction of compounds, while coarser grinds reduce the extraction due to the reduced contact area. These counter-intuitive results can be explained because the indirect immersion method was used in the cold extraction trials. In this method, the coffee grounds were placed inside a filter bag that was then placed in a vessel with water for the established extraction time. At the end of the trials, a greater caking effect was evidenced in the filter bags with medium ground coffee in comparison with those where coarsely ground coffee was used. During the extraction, this caking effect could have affected the diffusivity, lowering the detected values of TDS, EY and TPC in the brewed coffee. Recent studies have established that particle size, size distribution, and the coffee packing bed are parameters that affect and determine the diffusion of solids during the coffee extraction process^48–50^. Furthermore, the particle size distribution also affects the permeability and, therefore, the diffusion and mass transfer phenomena^51^. Given that the particle size distribution is a factor that affects the permeability and diffusion of chemical compounds during the extraction process, this phenomenon will be studied in futures investigations of the cold brew methods.

The long extraction times (22 h) that are applied in cold extraction tended to increase the values of TDS, EY and TPC, which were even higher when coarse grinding was used. In the extraction process, temperature can be considered as the driving force that favours the extraction of the chemical compounds present in the coffee grounds. At higher temperatures, the kinetic energy of the water molecules is higher, leading to a faster removal of the numerous compounds present in the roasted coffee. Conversely, during cold coffee brewing, the temperature is low (less than room temperature); therefore, the extraction and diffusion of several compounds requires more time to compensate for the low temperature. This could explain the fact that the cold brewed coffee with longer extraction times exhibited EY, TDS, and TPC values close to or even higher than their hot counterparts.

In addition to the effect of grinding and extraction time, the type of coffee also had a significant effect on the pH and titratable acidity (TA). Measurements of pH quantify the concentration of aqueous hydrogen ions at the time of analysis, providing a metric for the quantity of deprotonated acid molecules in a sample. Total titratable acidity is a measure of all acidic protons in a sample, including non-dissociated protons^27^. All cold brew samples evaluated in this study showed lower TA values than their hot counterparts. Recently, similar results have been reported by Rao & Fuller^27^ in cold brewing with coffees from different countries. These differences suggest that the cold and hot brew coffees are similar in their total concentrations of deprotonated acid compounds, but differ in the concentration, and possibly in the complexity, of protonated acids at the pH of extraction^27^. Several acids such as acetic, formic, malic, lactic and phosphoric, along with quinic and chlorogenic acids, have been detected in coffee brews^52^. Some of these can be extracted very efficiently in the initial seconds because their solubility increases with temperature, which could be why the hot brews exhibited higher TA values than the cold brews.

Although the coarse grinding and longest extraction time were identified as the optimal conditions for the physicochemical properties, higher scores in the global sensorial evaluation were reported for samples processed using coarse grinding and the lowest extraction time (14 h). This may be because lengthy extraction times can lead to over-extraction of the compounds associated with undesirable sensory notes. Likewise, lengthy extraction times may lead to the degradation of compounds in the beverage, and off-flavour compounds may be formed in the cold brew coffees. Illy and Viani^52^ found that in hot coffee brewing, over-extraction incorporated undesirable and less soluble aromatic compounds into the drink.

Several coffee vendors have marketed cold brew coffee with claims related to tasting smoother and less bitter and having a sweet taste and low acidity^27^. However, there are few published scientific studies that have carried out a cold brew flavour analysis. The results of this work revealed that cold brew coffees present global flavour profiles with less intensity in the aroma, taste, aftertaste, acidity, and body than their hot counterparts. The global perception or overall impact indicated that the assessors found that cold brew coffees had an acceptable flavour. The complexity of the cold brew coffee flavour also showed that there are specific attributes that are different from a hot brew. Cold brews presented the highest or similar values when compared to their hot counterparts for the flavour attribute scores of malt, pepper, almond, cocoa, chocolate, floral, bitterness, sweetness, and smooth and creamy body. Many of these had higher scores when a 14 h cold extraction was employed.

According to volatile profile results, both coffees presented differences in the number of compounds identified. As yet, there are no studies regarding the volatile compounds of cold brew coffees that allow making a rigorous comparison. In general, the high relative concentration of furans, pyrazines, and ketones in both samples (Nariño and Huila) could be associated with sweetness, malty and fruity, which was also described by the assessors in the sensorial analysis. Previous studies have indicated that the aroma of the filtered beverage is associated with the aromas of the alkylpyrazines, phenols, furanones and some thiols. These volatile compounds have been generally associated with sweet, caramel, herbal, fruity, malty, and nutty flavors^38^.

## Conclusions and Future Work

This research reports that some physicochemical characteristics, such as extraction yield, total dissolved solids, total phenolic content, pH and titratable acidity, can be strongly affected by the grinding level of coffee beans. When studying the role of grinding in the chemical composition of cold brew coffee, it is essential to consider the particle size distribution and coffee packing bed and their complex relationship with the diffusion and mass transfer phenomena. In cold extraction, a long contact time (22 h) increased the extraction yield, total dissolved solids, total phenolic content and titratable acidity. The type of coffee mainly affected the titratable acidity and pH. The Nariño cold brew samples showed titratable acidity values higher than those of Huila. Overall, all cold brew coffee treatments showed lower values of titratable acidity than their hot brew counterparts. The general characteristics of the cold brew flavour were less intense than those of their hot brew counterparts. According to the maximum overall sensory impression during the entire tasting time, the cold brewed coffee flavour was typical for this type of beverage.

The cold brew volatile profile identified in both coffees was related to some flavour attributes found in the sensorial analysis (sweetness, malty and fruity flavours). These results open new insights for future investigations related to the identification and quantification of volatile and non-volatile compounds and their relationship to the cold brew flavour profile under different extraction conditions.

## Methods

### Coffee

Arabica coffee (*Coffea arabica* var. *caturra*) from two origins, Huila and Nariño (Colombia), were used in all trials. The coffee beans corresponded to a micro-lot, which was harvest and processed by smallholder coffee growers in 2017. The roasted coffee beans from the Huila and Nariño regions of a medium-roasted degree were sourced from the local coffee store Cafecultor^®^ (Bogota, Colombia). The roasted coffee beans were ground in a commercial mill (BUNN^®^, Mexico D.F.) at two levels: coarse and medium. The particle size distributions of each grinding level were determined according to NTC 2441, Colombian Technical Standard, where the average of the particle size was determined by granulometric distribution^53^. An average particle size of 501 to 700 μm was used in medium grinding and 701–900 μm in the coarse grinding.

### Cold brew and hot brew experiments

The cold brewing process was carried out at room temperature (20 °C) using the immersion method with filtered water, according to Fuller and Rao^17^, with certain modifications. The coffee/water ratio used in all cases was 60 g ground coffee per 700 g water^17^. The effects of the particle size (coarse or medium), extraction time (14 or 22 h), and type of specialty coffee (Huila or Nariño) were studied. The experiments were carried out following a randomized design with a factorial structure (2 × 2 × 2) in duplicate. The measurements were carried out in triplicate. The extracts were stored in amber glass bottles at 4 °C until analysis. Hot brew extraction was performed according to Gloess, *et al*.^9^, with certain modifications; 42.5 g of coffee in 500 g of water was used to reach a coffee/water ratio similar to that in the cold brewing. French press coffee was prepared by pouring boiling water onto coffee grounds in a glass French press (Bodum^®^).

### TDS and EY

The TDS were measured following the methodology proposed by Moreno, *et al*.^54^, where defines the relationship between the concentration of soluble solids (X_s_) and the Brix degrees as follow: X_S_ = 0.0087 °Brix^54^. The EY was calculated as the relationship between the TDS, total weight of extract obtained (W_b_), and ground coffee weight used in the extraction (W_gc_), defined by the following equation^5^: EY (%) = (TDS * W_b_/W_gc_) * 100.

### Total TA and pH measurement

The pH value of the cold brew samples was measured at 19 °C using a pH-meter (Fisher Scientific, Ottawa, Canada). For the total TA, 50 mL of the coffee extract was titrated with 0.1 mol/L NaOH solution at a pH of 6.5, and the result was expressed in milligrams of chlorogenic acids per gram of coffee (NTC 5247)^55^.

### TPC

TPC measurements were conducted by means of the Folin–Ciocalteu method described by Singleton and Rossi^56^, with certain modifications^57^. Each sample was diluted 1:100 with distilled water. Thereafter, 90 µL distilled water, 15 µL sample, 7,5 µL Folin–Ciocalteu reagent (Sigma Aldrich, US), and 22.5 µL of 7.5% w/v sodium carbonate were added to each microplate, followed by the addition of 15 µL distilled water. All samples were incubated for 2 h at room temperature before measuring the absorbance at 750 nm. Each sample was measured in triplicate. The results were expressed in grams of gallic acid equivalents per liter of solution.

### Sensory evaluation of cold and hot brew coffees

Sensorial analysis of the coffee samples was carried out by a trained panel of three tasters (one female and two males), with more than three years of experience in the area of coffee sensory analysis. All judges had a broad experience and training in the cupping process established the SCAA protocol^36^ for coffee quality. Likewise, the panel selection was conducted according to requirements listed in ISO 8586-1^58^. Prior to the assessment of the cold brew coffee samples, the assessors participated in additional sessions to develop a sensory vocabulary for the cold coffee beverages. These samples were composed according to the Colombian Technical Standard NTC 3932^59^ and ISO 11035^60^. The initial list of attributes was classified into six categories: aroma, taste, aftertaste, acidity, body/mouthfeel, and overall impact. In each category, sensory descriptors were included according to those described by Chambers *et al*.^35^, with certain modifications. The tasters were trained for more than 40 h in cold brew coffee tasting, with the purpose of increasing their experience in the use of the vocabulary.

During the first stage, the sensory features of the cold and hot coffee samples were evaluated on a zero to five scale, using only the general groups of sensory descriptors (aroma, taste, aftertaste, acidity, body/mouthfeel, and overall impact). Within the scale, 3 corresponded to an attribute that was considered to be good, 3.5 was very good, 4 was excellent, and above 4.5 was outstanding. The panellists then evaluated the intensity of the specific attributes in each category. According to the range, five (5) was equivalent to a very strong attribute intensity, while zero (0) was not perceptible (NTC 3932, 1996)^59^. Each cold brew coffee sample was served in three replicates and was tested at room temperature (19 °C). The hot brews were assessed at 70 °C^61^, also in triplicate. The experimental design was made in two blocks, and the sessions for sensory analysis were performed on different days. The samples were analysed immediately after the completion of the extraction time. The cold and hot brews were evaluated in different sessions, and the sample order was randomized within each session and block.

### Volatile compound analysis

The volatile compounds of the cold brew coffee were obtained by Headspace–Solid-Phase Microextraction (HS-SPME) and analyzed by gas chromatography coupled with mass spectrometry (GC–MS), according to the method reported by Moreno, *et al*.^62^, with certain modifications. Each sample (2 g) was equilibrated for 1 h in a 40 mL sealed vial at 18 °C using a magnetic stirrer. The volatile compounds released from the headspace of each sample were collected using 65 µm PDMS/DVB 1 cm long fiber (Supelco Inc., Bellefonte, PA, USA) for 1 h at 18 °C and then directly injected (5 min of fiber desorption time at 250 °C) into a gas chromatograph, Agilent 7890B, coupled to a 5977A mass selective detector (Agilent Technologies Inc., Wilmington, DE, USA). A DB-FFAP column (Agilent Technologies Inc., Wilmington, DE, USA, 50 m × 0.25 mm i.d., 0.32 μm) was used. The column oven was programmed from 45 (after 2 min) to 250 °C at 5 °C/min, and the final temperature was maintained for 5 min. The injector temperature was maintained at 250 °C; the carrier gas was 1.5 mL of He/min. Structural elucidation of the volatile compounds was performed by comparison of their mass spectra and retention indexes (retention index using a C_8_–C_26_ alkane standard mix), with those of either the standards or NIST mass spectra library (ver. 2.2, NIST/EPA/NIH, 2014). Data were processed by the Class 5000 v 2.2 MS-Workstation software.

### Ethical permission

All experiments were carried out following relevant guidelines and regulations of Universidad de La Sabana. The Research Commission of the Faculty of Engineering of the Universidad de La Sabana approved the procedures and methodologies related to the sensory analysis of the coffee extracts. All participants of the sensory analysis were volunteers and signed an informed consent form with the type of the research and agreement to develop the sensory analysis of the coffee extracts produced under the conditions described in the section “sensory evaluation of cold and hot brew coffee.”

### Data analysis

The effects of the particle size, extraction time, and coffee origin on the physicochemical variables and sensory attributes were evaluated using a randomized block factorial design. One-way analysis of variance was applied to determine the difference between treatments, followed by an LSD test with a significance level of 95% using XLStat version 2018.1 (Addinsoft Corp., France).
